# Premenstrual symptoms and academic performance: exploring female medical students' experience, triggering factors, and coping approaches

**DOI:** 10.3389/fgwh.2026.1758225

**Published:** 2026-06-18

**Authors:** Eman Dawood, Shahad Alshareif, Hend Binghadir, Sharifah Alotaibi, Deem Alofisan, Hanaa M. Abo Shereda

**Affiliations:** 1 College of Nursing, King Saud Bin Abdulaziz University for Health Sciences, Riyadh, Saudi Arabia; 2 King Abdullah International Medical Research Center, Riyadh, Saudi Arabia; 3 Department of Psychiatric and Mental Health Nursing, College of Nursing, Menofyia University, Shebin-Elkom, Egypt; 4 Ministry of the National Guard - Health Affairs, Riyadh, Saudi Arabia; 5 College of Medicine, King Saud Bin Abdul-Aziz University for Health Sciences, Riyadh, Saudi Arabia

**Keywords:** coping strategies, medical students, menstrual health, premenstrual dysphoric disorder, premenstrual syndrome

## Abstract

**Background:**

Premenstrual dysphoric disorder (PMDD) represents the most severe form of premenstrual symptoms, markedly affecting emotional well-being, physical health, and academic functioning. Medical students are particularly susceptible due to high academic demands and stress exposure. However, evidence from Saudi Arabia remains limited. This study examined the proportion of participants who screened positive for PMDD based on PTSS criteria, its associated triggering factors, and coping strategies among female medical students in the central region of Saudi Arabia.

**Methods:**

A cross-sectional descriptive study was conducted among 492 female students enrolled in medical colleges. Participants were recruited through convenience sampling. Data were collected using a self-administered questionnaire consisting of sociodemographic and menstrual-related items and the validated Premenstrual Symptoms Screening Tool (PSST). Data analysis was performed using JMP Pro 17. Ethical approval was obtained from the Institutional Review Board at KAIMRC (SP23R/242/11). The study adhered to STROBE reporting guidelines.

**Results:**

The proportion of participants who screened positive for PMDD based on PTSS criteria of PMDD was 11%, while 42.5% of participants met criteria for moderate-to-severe premenstrual syndrome (PMS). The most frequently reported symptom triggers included stress (70.33%), poor general health (43.90%), poor nutrition (38.82%), and academic examinations (38.62%). Common coping strategies included rest (70.33%), consumption of warm herbal drinks (49.59%), and the use of heat therapy (48.58%). Only 21% reported seeking medical consultation. Significant associations were identified between PMS/PMDD severity and marital status (*p* = 0.010), college type (*p* = 0.029), menstrual blood amount (*p* < 0.001), dysmenorrhea (*p* < 0.001), and menstrual cycle regularity (*p* < 0.001). Symptom severity demonstrated a strong positive correlation with absenteeism due to dysmenorrhea (r = 0.402, *p* < 0.001).

**Conclusion:**

PMDD and moderate-to-severe PMS are common among female medical students and are influenced by stress, lifestyle factors, and menstrual health characteristics. Despite the considerable impact on academic functioning and attendance, most students adopt self-management approaches and rarely seek medical care. These findings underscore the need for targeted health education, stress management support, and enhanced menstrual health services within academic institutions.

## 1 Background

Premenstrual dysphoric disorder (PMDD) represents a severe clinical form of premenstrual syndrome (PMS) and is recognized in the Diagnostic and Statistical Manual of Mental Disorders, fifth edition, Text Revision (DSM-5- TR) as a distinct psychiatric condition. According to DSM-5-TR criteria, PMDD is characterized by recurrent physical, emotional, and cognitive symptoms that occur during the luteal phase of the menstrual cycle and significantly impair social, academic, and occupational functioning (31). In the updated DSM-5-TR (2022), PMDD is classified under depressive disorders, with emphasis on prospective daily symptom ratings across at least two symptomatic cycles to confirm the diagnosis. Common symptoms include mood lability, irritability, depressed mood, anxiety, somatic complaints, and cognitive disturbances (1).

Premenstrual syndrome (PMS) and premenstrual dysphoric disorder (PMDD) are related but distinct conditions. PMS includes a range of physical, emotional, and behavioral symptoms that occur before menstruation and are generally mild to moderate in severity. In contrast, PMDD represents a more severe and clinically significant form, characterized by pronounced mood disturbances and functional impairment as defined in the DSM-5- TR (31).

Evidence suggests that female medical students represent a particularly vulnerable population and are disproportionately affected by PMS, with symptoms ranging from mild discomfort to severe manifestations that interfere with daily functioning compared to paramedical students, due to the high academic and psychological demands of their training (2). The World Health Organization estimates that approximately 20%–31% of female university students worldwide experience menstruation-related mental health problems (3, 4). PMS is increasingly recognized as a public health concern, particularly among younger populations (5). Prevalence rates among medical students are notably high, reaching up to 65% in some studies (6). In Saudi Arabia, Rafique & Al-Sheikh (7) reported a high prevalence of menstrual disorders and stress among female health sciences students, with significant associations between psychological stress and conditions such as amenorrhea, dysmenorrhea, and PMS.

The impact of PMDD and PMS on academic and social functioning is substantial. Studies have demonstrated negative associations with academic performance, reduced participation in social activities, and increased absenteeism (6). For example, research conducted at Mekelle University found that 83.2% of students reported at least one PMS symptom, with 37% meeting PMS criteria; these symptoms were linked to missed classes, decreased academic performance, and occasional withdrawal from coursework (8). Similarly, dysmenorrhea has been associated with reduced classroom engagement, impaired concentration, and declining academic outcomes (9, 30). Somatic symptoms, particularly body discomfort, and affective symptoms such as irritability are among the most commonly reported (6).

Lifestyle and behavioral factors play a critical role in the severity and occurrence of PMS/PMDD. Research from El-Minia University identified several associated risk factors, including family history, physical inactivity, low body mass index, excessive caffeine consumption, and smoking (10). Additional studies have linked PMS to poor dietary habits, sleep disturbances, and menstrual pain severity (11, 12, 29). The COVID-19 pandemic has further exacerbated menstrual health disturbances. Stress associated with the pandemic has been shown to disrupt glucocorticoid levels, thereby affecting menstrual cycle regularity (13). Subsequent studies reported alterations in menstrual patterns, including cycle irregularity and worsening PMS symptoms (14). In Saudi Arabia, recent findings from Jeddah indicated that 61.5% of women experienced increased menstrual pain and changes in premenstrual symptoms during the pandemic, largely attributed to psychological stress (15). Similarly, research from King Saud University found that 46.9% of female students reported menstrual irregularities following COVID-19 infection or vaccination (16).

Despite the high prevalence and impact of PMDD, help-seeking behavior among students remains low. Many individuals avoid seeking medical care due to stigma or the perception that symptoms are a normal part of menstruation (17). Instead, students frequently rely on self-medication or informal coping strategies (18, 19). In Saudi Arabia, approximately 98% of individuals with PMS report using self-management approaches (20). A recent study at Princess Nourah University found that 39.9% of female students met PMDD criteria; however, many relied on non-medical coping strategies, including ignoring symptoms or using over-the-counter medications without professional consultation (21). These findings highlight the need for improved awareness and access to appropriate healthcare services.

Given the demanding nature of medical education, premenstrual symptoms may significantly compromise students' academic performance and overall well-being. Identifying contributing factors and effective coping mechanisms is essential for developing supportive interventions. Increasing awareness, promoting healthy lifestyle behaviors, and ensuring access to medical and psychological support services are critical steps in mitigating the impact of PMDD. Furthermore, educational institutions should consider implementing policies and accommodations that address the specific needs of affected students. As emphasized by Kamel et al. (22), integrating stress management programs, accessible healthcare services, and targeted educational initiatives can foster a more supportive and inclusive academic environment. Ultimately, addressing PMDD is essential not only for improving health outcomes but also for enhancing academic success among female medical students.

Despite extensive research on PMS and PMDD, limited evidence integrates the effects of psychological stress, lifestyle factors, and post-pandemic influences on PMDD severity among medical students (27, 28). Moreover, the relationship between PMDD symptom severity and academic performance remains insufficiently quantified, particularly in the Saudi context where research has largely focused on prevalence rather than functional impact. Additionally, the effectiveness of coping strategies and support systems among affected students still underexplored.

## 2 Materials and methods

This study aims to investigate the impact of premenstrual dysphoric disorder on academic performance among female medical students, with a focus on associated risk factors and coping strategies in the central region of the Kingdom of Saudi Arabia on a sample of 492 female medical students who met the inclusion criteria and recruited via convenience sampling technique. We employed a cross sectional descriptive research design to conduct the study. Data was collected using two parts self-administered anonymous English questionnaire survey.

Part one is the demographic data sheet, which was developed by the researchers. It included age, marital status, year of study, college, age of menarche, number of average days bleeding per one cycle, dysmenorrhea, severity of menstrual pain, sources of information about PDD, triggering factors and ways of coping with the menstrual pain.

Part two was the English version of the Premenstrual Symptoms Screening Tool (PSST) developed by Steiner et al. (23), which was used to assess the presence and severity of premenstrual symptoms after obtaining the proper approvals and permissions to utilize the tool in the current study. It consists of 19 items, 14 premenstrual symptoms, and 5 functional items, in line with Diagnostic and Statistical Manual (DSM-5) criteria. The PSST is a self-assessment tool with four possible answers, the severity of which is rated using a 4-point Likert scale, with 1 = not at all, 2 = mild, 3 = moderate and 4 = severe. Participants were asked to rate the extent to which they experience each symptom during the late luteal phase (the week before bleeding) and stopping within a few days of bleeding and the extent to which the symptoms interfere with each functional domain (work efficiency or productivity; relationships with co-workers; relationship with family; social life activities; home responsibilities). Internal consistency were evaluated by using Cronbach's alpha. The value of this index for the symptoms was 0.90, and the impact of symptoms on life was 0.91 and the total questions was 0.93 (24). The scale is available and validated into many languages.

Collected data was analyzed using JMP Pro17. Categorical variables were presented as frequencies and percentages. Continuous variables were presented as means and standard deviations. The chi-squared test was used to compare categorical variables. Associations between continuous variables (age, student' GPA, Age of menarche, number of annual college absenteeism days due to dysmenorrhea) and the total PSST score were examined using correlation analysis. Correlation coefficients (r) and corresponding *p*- value were calculated to assess the strength and significance of these relationships. A *P*- value of <0.05 was considered statistically significant. Review and approval were obtained from the research unit at the College of Nursing, KSAU-HS. Ethical approval was granted through the Institutional Review Board Committee (IRB) at KAIMRC (IRB/0133/24). This study adhered to the Strengthening the Reporting of Observational Studies in Epidemiology (STROBE) guidelines.

## 3 Results

Four hundred and ninety two female students from different medical colleges and different academic levels participated in the study (Table 1). Participants' age ranged between 18 and 38 years with a mean age of 21.58 + 2.33 year. The vast majority were singles (*n* = 436, 88.62%) while only 56 (11.38%) were married. Duration of study in their concerned college ranged from a minimum of 1 to a maximum of 7 years with a mean of 3.34 + 1.42 years studying in the college. Students' GPA ranged between 2 and 5 with a mean GPA score of 4.11 + 0.63. Majority of the students were from nursing college (30.8%) followed by college of dentistry (25.2%), college of medicine (16.6%), college of science and health professions (15.8%) and. Their calculated body mass index ranged between 13.97 and 40.74 with a mean body mass index of 22.79 + 4.80. Following the WHO Adult BMI Categories classification, almost two thirds of the participants (291, 59.15%) were in the normal BMI category, while 78 (15.8%) were overweight and only 47 (9.5%) were obese. Age of menarche ranged between 8 and 17 years with a mean of 12.71 + 1.65 years. Number of average days bleeding per one cycle in the last year reported to range from two to twelve days with a mean of 6.0 + 1.76 days.

**Table 1:** Sociodemographic characteristics of the study sample (*n* = 492).

Variable	Frequency (*N*)	Percent (%)
Age: 18–38 years		
Mean 21.58		
SD ± 2.33		
Marital Status		
Single	436	88.6
Married	56	11.4
College		
College of Science and Health Professions (COSHP)	78	15.85
Medicine	82	16.66
Nursing	152	30.89
Dentistry	124	25.20
Pharmacy	32	06.50
Public Health	10	02.03
Applied Medical Sciences	14	02.84
The amount of blood during one menstrual period		
Mild	84	17.07
Moderate	298	60.56
Heavy	94	19.10
Extremely Heavy	16	03.3
Dysmenorrhea		
Not at all	92	18.69
Mild	174	35.36
Moderate	142	28.86
Severe	84	17.07
Menstrual cycle		
Regular	384	78.05
Irregular	108	21.95

Data are presented as number (percentage) for categorical variables and as mea*n* ± standard deviation (SD) and range for age.

Number of annual college absenteeism in days due to dysmenorrhea was reported by the participants to range between a minimum of zero and a maximum of 35 with a mean of 4.11 ± 5.29 days per academic year.

Majority of the participants reported the amount of blood during one menstrual period to be moderate (*n* = 298, 60.6%) followed by heavy (*n* = 94, 19.1%) and only sixteen (3.3%) reported extremely heavy bleeding. More than three quarters of the participants (78.1%) reported their menstrual cycle as regular while only 21.9% reported irregular menstrual cycles, consequently 79.3% never consulted with a physician because of menstrual related concerns while only 102 (20.7%) visited a physician due to menstrual related concerns. 84 (17%) reported severe dysmenorrhea while 18% reported no dysmenorrhea at all. Participants were asked to rate on a numerical scale, how do they rate the severity of menstrual pain from zero (no pain) to 10 (the worst pain imaginable), the mean pain score was 5.93 ± 2.5. 57.7% had information about premenstrual dysphoric disorder while 42.3% did not know about the disorder.

Table 2 illustrates the distribution of sources from which respondents obtained information about Premenstrual Dysphoric Disorder (PMDD). The data indicate that school or college classes (61.79%) and the internet (44.30%) are the primary sources of information about Premenstrual Dysphoric Disorder (PMDD) among respondents, highlighting the crucial role of formal education and digital media in health awareness. In contrast, informal sources such as family (8.94%) and friends (8.53%), as well as traditional media (5.28%) and printed materials (5.07%), were cited less frequently. the data indicate that structured educational environments and digital media are the primary sources through which females acquire knowledge about PMDD. The relatively lower reliance on interpersonal and traditional media sources may reflect gaps in informal health communication and the need for broader community-based awareness strategies about PMDD.

**Table 2:** Source of information about premenstrual dysphoric disorder (*n* = 492).

Source of Information	Frequency (*N*)	Percent (%)
School or college classes	304	61.79
Internet	218	44.30
Family	44	8.94
Friends	42	8.53
Media including TV, Radio	26	5.28
Books, magazines or journals	25	5.07
Others	9	1.83

Participants can select more than one option from all options provided.

Table 3 illustrates the perceived triggering factors of premenstrual symptoms as reported by the study participants. The majority of participants identified stress as the most common trigger of premenstrual symptoms (70.33%), followed by poor general health (43.90%), poor nutrition (38.82%), and academic examinations (38.62%). Other frequently reported factors included lack of sleep (36.58%). A smaller proportion of respondents attributed their symptoms to unknown causes (19.92%) or expressed uncertainty about the triggers (10.77%). These findings suggest that both lifestyle and psychosocial factors play a significant role in the experience of premenstrual symptoms, underscoring the need for greater awareness and management strategies focused on stress, health maintenance, and lifestyle balance.

**Table 3:** Triggering factors of premenstrual symptoms (*n* = 492).

Premenstrual symptoms trigger factors	Frequency (*N*)	Percent (%)
1. Stress	346	70.33
2. Poor general health	216	43.90
3. Poor nutrition	191	38.82
4. Academic examinations	190	38.62
5. Lacking sleep	180	36.58
6. Unknown cause	98	19.92
7. I do not know	53	10.77

Participants can select more than one option from all options provided.

Table 4 presents the various management approaches reported by participants in addressing premenstrual symptoms. The most commonly adopted strategy was rest, utilized by 70.33% (*n* = 346) of respondents, emphasizing the perceived benefit of physical relaxation in symptom relief. The use of herbal remedies in the form of warm fluids—such as mint, cinnamon, lemon, or chamomile—was the second most frequent approach, reported by 49.59% (*n* = 244), reflecting a reliance on natural and culturally familiar remedies. Additionally, thermal therapies, such as taking a hot shower or using a hot bag, were employed by 48.58% (*n* = 239), while sleeping was another common coping strategy, reported by 40.04% (*n* = 197). These non-pharmacological approaches indicate a preference for self-care and lifestyle modifications among a large portion of the sample. Pharmacological interventions were less frequently used. Over-the-counter oral analgesics such as Panadol, Ibuprofen, or Voltron were taken by 35.78% (*n* = 176) of participants. More intensive medical interventions, including intramuscular analgesics (5.69%, *n* = 28), intravenous analgesics (3.45%, *n* = 17), and prescribed medications (3.86%, *n* = 19), were reported by a minority. Only 3.25% (*n* = 16) of respondents reported seeking emergency health services for symptom management. Further, 21% of our participants reported consulting with a physician because of the premenstrual symptoms, while the majority 79% never had a consultation with a specialist with regard to any of the premenstrual symptoms.

**Table 4:** Premenstrual symptoms management approaches as reported by the study participants (*n* = 492).

Management Approache	Frequency (*N*)	Percent (%)
1. Rest	346	70.33
2. Herbs in the form of warm fluids like mint, cinnamon, lemon, chamomile	244	49.59
3. Taking hot shower or using hot bag	239	48.58
4. Sleeping	197	40.04
5. Taking over the counter oral analgesics as Panadol, Ibuprofen, Voltron	176	35.78
6. Intramuscular analgesics	28	5.69
7. Intravenous analgesics	17	3.45
8. Prescribed medication	19	3.86
9. Seeking emergency health services	16	3.25

Participants can select more than one option from all options provided.

Overall, the findings suggest that while a majority of participants relies on rest and home-based or natural remedies for managing premenstrual symptoms, a smaller proportion resort to pharmacologic or clinical interventions. This may reflect the self-limiting nature of symptoms for many individuals, cultural preferences, or potential barriers to accessing professional healthcare services.

Table 5 presents the frequency and severity of premenstrual symptoms experienced by participants based on the Premenstrual Symptoms Screening Tool (PSST), along with the impact of these symptoms on various areas of functioning. The PSST results show that a significant proportion of participants experienced moderate to severe premenstrual symptoms, particularly anger/irritability (44.71%), anxiety (47.15%), depressed mood (50.61%), and fatigue (59.75%). Physical symptoms and concentration difficulties were also common. These symptoms influenced daily life, with notable interference in work productivity (45.53%), social activities (43.69%), and home responsibilities (33.94%). The findings underscore the substantial psychological and functional burden of premenstrual symptoms among respondents.

**Table 5:** Participants responses to the premenstrual symptoms screening tool (PSST) (*n* = 492).

Symptom	Not at all	Mild	Moderate	Severe
1. Anger/irritability	79 (16.06)	193 (39.23)	158 (32.11)	62 (12.60)
2. Anxiety/tension	81 (16.46)	179 (36.38)	167 (33.94)	65 (13.21)
3. Tearful/sensitive to rejection	124 (25.20)	172 (34.96)	133 (27.03)	63 (12.80)
4. Depressed mood/hopelessness	70 (14.23)	173 (35.16)	150 (30.49)	99 (20.12)
5. Decreased interest in work activities	76 (15.45)	186 (37.81)	145 (29.48)	85 (17.28)
6. Decreased interest in home activities	68 (13.82)	198 (40.24)	125 (25.41)	101 (20.53)
7. Decreased interest in social activities	84 (17.07)	175 (35.57)	148 (30.08)	85 (17.28)
8. Difficulty concentrating	98 (19.92)	198 (40.24)	123 (25.00)	73 (14.84)
9. Fatigue/lack of energy	44 (08.94)	154 (31.30)	170 (34.55)	124 (25.20)
10. Overeating/food cravings	104 (21.14)	161 (32.72)	141 (28.66)	86 (17.48)
11. Insomnia	192 (39.02)	147 (29.88)	84 (17.07)	69 (14.02)
12. Hypersomnia	188 (38.21)	121 (24.59)	104 (21.14)	79 (16.06)
13. Feeling overwhelmed or out of control	112 (22.76)	161 (32.72)	126 (25.61)	93 (18.90)
14. Physical symptoms	60 (12.20)	184 (37.40)	152 (30.89)	96 (19.51)
Have your symptoms listed above interfered with your functioning?				
A. Work efficiency/ productivity	80 (16.26)	188 (38.21)	173 (35.16)	51 (10.37)
B. Relationship with co-workers	163 (33.13)	200 (40.65)	108 (21.95)	21 (04.27)
C. Relationship with family	132 (26.83)	193 (39.23)	126 (25.61)	41 (08.33)
D. Social life activities	104 (21.14)	173 (35.16)	180 (36.58)	35 (07.11)
E. Home responsibilities	116 (23.58)	209 (42.48)	132 (26.83)	35 (07.11)
Total PSST score ranged between 0 and 55 (25.81 + 11.88)				

PSST items are rated on a 4-point Likert scale (1 = Not at all to 4 = Severe). Data are presented as Frequency and percentages. The overall PSST score is expressed as mean ± standard deviation (SD).

The total PSST score ranged between zero and 55 with a mean scale score of 25.81 ± 11.88. The symptoms subscale mean score was 19.82 + 9.13 with a minimum subscale score of zero and a maximum score of 42. With regard to the effect of the symptoms, the subscale scores ranged between zero and 15 with a mean of 5.98 + 3.52.

Based on the Premenstrual Symptoms Screening Tool (PSST) scoring guidelines, analysis of our data indicated that 11% (*n* = 59) of participants screened positive for probable PMDD, (PMDD), 42.5% (*n* = 209) met the criteria for moderate-to-Severe premenstrual syndrome (PMS). The remaining 45.5% (*n* = 224) did not meet the PSST criteria for either condition.

The association between selected demographic, academic, and menstrual-related variables and the severity of premenstrual symptoms as categorized by the Premenstrual Symptoms Screening Tool (PSST) among a sample of 492 female medical students is presented in Table 6, Marital status was significantly associated with PSST categories (*χ*² = 6.431, *p* = 0.010), with single students more likely to report moderate-to-severe PMS and PMDD than married students. This suggests marital status may influence the perception or reporting of premenstrual symptoms. College affiliation also showed a significant association (*χ*² = 20.899, *p* = 0.029), with Nursing and Dentistry students reporting higher rates of PMS and PMDD, possibly due to greater academic or clinical stress and heightened health awareness. Additionally, a strong association was observed between the amount of menstrual bleeding and PSST categories (*χ*² = 47.740, *p* < 0.001), with heavier bleeding linked to more severe symptoms. Dysmenorrhea was also significantly related (*χ*² = 81.811, *p* < 0.001), as students with moderate to severe pain reported higher rates of PMS and PMDD. Finally, menstrual cycle regularity was significantly associated with PSST categories (*χ*² = 17.635, *p* < 0.001), with irregular cycles linked to increased risk of PMDD, highlighting a potential clinical indicator of symptom severity.

**Table 6:** Relationship between medical students’ selected variables and PSST categories (*n* = 492).

Variable	Unlikely to have PMD (*N*, %)	Moderate-to-Severe PMS (*N*, %)	PMDD (*N*, %)	*χ*2	*P*-Value
Marital Status					
Single	197 (40.0)	181 (36.8)	58 (11.8)	6.431	0.010*
Married	27 (5.5)	28 (5.7)	1 (0.2)		
College					
COSHP	45 (9.2)	27 (5.5)	6 (1.2)		
Medicine	31 (6.3)	39 (7.9)	12 (2.4)		
Nursing	74 (15.0)	53 (10.8)	25 (5.1)	20.899	0.029*
Dentistry	50 (10.2)	62 (12.6)	12 (2.4)		
Pharmacy	14 (2.9)	16 (3.3)	2 (0.4)		
Public Health	2 (0.4)	6 (1.2)	2 (0.4)		
Applied Medical Sciences	8 (1.6)	6 (1.2)	0 (0.0)		
Body Mass Index (BMI)					
Underweight	29 (5.9)	38 (7.7)	9 (1.8)		
Normal	130 (26.4)	127 (25.8)	34 (6.9)	5.359	0.498
Overweight	39 (7.9)	28 (5.7)	11 (2.2)		
Obese	26 (5.3)	16 (3.3)	5 (1.0)		
Information about PMDD					
Yes	137 (27.9)	115 (23.4)	32 (6.5)	2.002	0.367
No	87 (17.7)	94 (19.1)	27 (5.5)		
Amount of Menstrual Blood					
Mild	55 (11.2)	27 (5.5)	2 (0.4)		
Moderate	136 (27.6)	131 (26.6)	31 (6.3)	47.740	0.000*
Heavy	30 (6.1)	46 (9.4)	18 (3.7)		
Extremely Heavy	3 (0.6)	5 (1.0)	8 (1.6)		
Dysmenorrhea					
Not at all	64 (13.0)	23 (4.7)	5 (1.0)		
Mild	97 (19.7)	68 (13.8)	9 (1.8)	81.811	0.000*
Moderate	51 (10.4)	71 (14.4)	20 (4.1)		
Severe	12 (2.4)	47 (9.6)	25 (5.1)		
Menstrual Cycle Regularity					
Regular	186 (37.8)	164 (33.3)	34 (6.9)	17.635	0.000*
Irregular	38 (7.7)	45 (9.2)	25 (5.1)		

PMD, Premenstrual disorders; PMS, Premenstrual syndrome; PMDD, Premenstrual dysphoric disorder, COSHP, College of Science and Health Professions.

* *P*-values marked with an asterisk indicate statistical significance at *p* < 0.05.

Conversely, prior knowledge of PMDD was not significantly related to PSST classifications (*χ*² = 2.002, *p* = 0.367), suggesting awareness alone does not influence symptom severity. Similarly, no significant association was found between BMI and symptom severity (*χ*² = 5.359, *p* = 0.498), indicating BMI may not be a key factor in premenstrual symptoms.

As illustrated in Table 7, Correlation analysis revealed no significant relationship between total PSST scores and participants' age (*r* = 0.001, *p* = 0.977) or their GPA (*r* = 0.056, *p* = 0.209). A weak but statistically significant negative correlation was observed between age at menarche and PSST scores (*r* = −0.099, *p* = 0.028), suggesting that earlier menarche is slightly associated with higher symptom severity.

**Table 7:** Relationship between medical students’ selected variables and PSST score (*n* = 492).

Variable	Total PSST Score	
	r (correlation coefficient)	*P* (*p*-value)
Age	0.001	0.977
Students’ GPA	0.056	0.209
Age of Menarche	−0.099	0.028*
Number of annual college absenteeism in days due to dysmenorrhea	0.402	0.000*

* *P*-values marked with an asterisk indicate statistical significance at *p* < 0.05.

On contrast, a strong association was detected between PSST scores and annual college absenteeism due to dysmenorrhea (*r* = 0.402, *p* < 0.001), indicating that higher symptom severity is linked to increased absenteeism from college.

## 4 Discussion

The results of this study indicated a higher proportion of participants who screened positive for PMDD based on PTSS criteria of PMS compared to PMDD. PMS was reported by 42.5% of participants, whereas PMDD was identified in 11%, which aligns with expectations since PMDD represents a more severe progression of PMS. However, the proportion of participants who screened positive for PMDD based on PTSS criteria observed in this study was lower than that reported in previous research by (6, 25, 26), who documented rates of 65%, 80.1%, and 69%, respectively. Differences in prevalence may be attributed to variations in study populations, timing, and measurement tools. Both PMS and PMDD were more prevalent among single students and those enrolled in nursing and dentistry programs, which are more clinically demanding. This finding is consistent with Thakrar et al. (2), who reported higher prevalence among medical students. Overall, PMS and PMDD remain significant health concerns among female medical students.

The findings also confirmed key triggers of premenstrual symptoms. Stress emerged as the most common trigger, reported by 70.33% of participants. This strong association between psychological stress and PMS has been documented in previous studies, including Rafique and Al-Sheikh (7). Academic examinations were also identified as a trigger, likely reflecting stress related to preparation and performance. Other notable factors included poor general health and inadequate nutrition, suggesting that interventions should address both psychological and lifestyle components.

Coping strategies reported by participants were predominantly nonpharmacological. Rest was the most common approach (70.33%), followed by herbal remedies (49.59%) and thermal therapies (48.58%). Only 21% of participants consulted a physician, and 35.78% used over-the-counter medications, indicating a strong preference for self-care and lifestyle-based interventions. These findings align with previous research by Tsegaye and Getachew (17), Dilbaz and Aksan (18, 19), which highlighted limited medical consultation and widespread self-medication among students. The reliance on nonpharmacological strategies suggests perceived effectiveness and cultural acceptability of these approaches.

Contrary to some previous studies (6, 8, 9), this study did not find a significant association between PMS or PMDD and academic performance as measured by GPA. However, GPA alone may not fully capture academic impact, as absenteeism and reduced engagement were not assessed in detail. Notably, a strong correlation was observed between symptom severity and absenteeism due to dysmenorrhea, indicating that menstrual symptoms can disrupt academic participation even if GPA remains unaffected.

## 5 Conclusion

This study highlights that PMDD and moderate-to-severe PMS are highly prevalent among female medical students in the central region of Saudi Arabia, with symptoms strongly influenced by stress, lifestyle factors, and menstrual health patterns. Despite the substantial impact on academic performance, daily functioning, and absenteeism, many students rely primarily on self-management strategies and seldom seek professional medical care. These findings emphasize the urgent need for targeted educational programs, accessible menstrual health services, and stress-reduction initiatives within academic settings. Strengthening awareness, early identification, and supportive interventions may significantly improve students' well-being, academic success, and overall quality of life.

### 5.1 Implications and limitations

These findings have practical implications. First, the strong link between stress and PMS underscores the need for stress management interventions, particularly for students in high-pressure academic environments. Second, the preference for nonpharmacological coping strategies suggests that health education programs should incorporate culturally relevant lifestyle approaches alongside medical options.

Despite these insights, the study has the following limitations: its cross-sectional design precludes causal inference, and convenience sampling limits generalizability. Self-reported data may also introduce recall bias. The lack of multivariable analysis to control for potential confounding factors. Future research should employ longitudinal designs and representative sampling to validate prevalence estimates and explore causal relationships using multivariable analysis (e.g., ordinal or multinomial logistic regression) to control for possible confounding variables and to provide a more robust understanding of these relationships. Additionally, experimental studies on the effectiveness of nonpharmacological interventions could inform evidence-based practice and health education initiatives.

## Data Availability

The original contributions presented in the study are included in the article/Supplementary Material, further inquiries can be directed to the corresponding author/s.
